# Pillar[6]arene-based supramolecular polymeric materials constructed *via* electrostatic interactions for rapid and efficient organic dye removal from water†

**DOI:** 10.1039/d0na00938e

**Published:** 2021-02-15

**Authors:** Xin Yan, Youyou Huang, Moupan Cen, Jin Wang, Jian Shi, Bing Lu, Yang Wang, Yong Yao

**Affiliations:** School of Chemistry and Chemical Engineering, Nantong University Nantong Jiangsu 226019 P. R. China yaoyong1986@ntu.edu.cn; Nantong University Analysis & Testing Center Nantong Jiangsu 226019 P. R. China shi.j1@ntu.edu.cn

## Abstract

The first pillar[6]arene-based supramolecular polymeric material constructed from electrostatic interactions was designed and prepared successfully. Importantly, it can adsorb and remove organic dye from water efficiently.

## Abstract 2

Pillar[6]arene-based polymeric materials were constructed from electrostatic interactions efficiently, and can further be applied in removing organic dyes from water.

## Graphical abstract

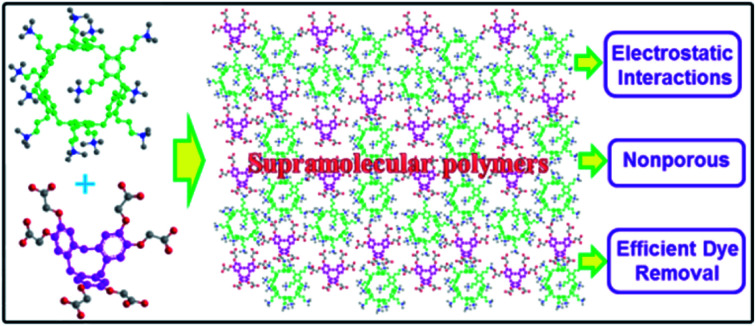

Organic dyes such as azophloxine, alcian blue, rhodamine B and so on are very important materials for the textile industry.^1^ However, the direct discharge of textile wastewater will cause serious water pollution and do harm to human health.^2^ To remove organic pollutants from water, adsorption with activated carbon (ATC) has been widely used due to its high efficiency and relatively low cost. But the regeneration of ATC, usually under air conditions with heating up to 500 °C, is energy intensive and will cause secondary pollution. Therefore, it is quite necessary to design and fabricate novel adsorbent materials with rapid adsorption and efficient uptake ability.^3^

Pillar[*n*]arenes,^4^ composed of hydroquinone derivative units and linked by –CH_2_– in the 2,5-positions, are a new type of emerging macro-cyclic host after crown ethers,^5^ cyclodextrins,^6^ calixarenes,^7^ and cucurbiturils.^8^ The facile syntheses, unique rigid pillar-like architectures, and π-electron rich cavities of pillar[*n*]arenes make them outstanding affinity hosts for selectively neutral and electron deficient guests.^9^ Considering the low preparation costs and the rich host–guest interactions, pillar[*n*]arene-based functional materials have been widely investigated and applied in various areas, such as drug delivery systems,^10*a*^ molecular machines,^10*b*^ trans-membrane channels^10*c*^ and supramolecular polymers.^10*d*^ Fortunately, adsorbent materials based on pillar[*n*]arene cross-linked polymers (AMPCPs) with rapid adsorption and efficient uptake ability have also been designed and fabricated in the past several years.^11^ For example, Prof. Ma and co-workers prepared a type of AMPCP by the reaction between tetrafluoroterephthalonitrile (TFTN) and hydroxyl units on pillar[*n*]arene for the efficient removal of organic pollutants from water.^11*a*^ Prof. Huang and co-workers produced another type of AMPCP for the adsorption and removal of organic dyes from water by cross-linking carboxyl-derived pillar[5]arene and *p*-phenylenediamine.^11*b*^ Prof. Coskun constructed a conjugated AMPCP incorporating pillar[5]arenes *via* a Pd-catalyzed Sonogashira–Hagihara cross-coupling reaction for propane/methane separation through host–guest interactions.^11*c*^

However, previous reported AMPCPs are all constructed by covalent bonds, and it is still a challenge to design and prepare AMPCPs from supramolecular interactions, such as electrostatic interactions, host–guest interactions and so on because supramolecular interactions are very weak and the AMPCPs from supramolecular interactions are not stable enough. Herein, we designed and constructed a novel AMPCP (WP6&WCTV) from quaternary ammonium modified water soluble pillar[6]arene (WP6) and carboxyl-derivatived water soluble cyclotriveratrylene (WCTV) *via* electrostatic interactions. The as-prepared WP6&WCTV polymeric materials were fully characterized by SEM, TEM, EDX mapping, solid-state ^13^C-NMR and elemental analysis. What's more, they can remove organic dye from water with a very fast adsorption rate and high adsorption capacity (Scheme 1).

**Scheme 1:**
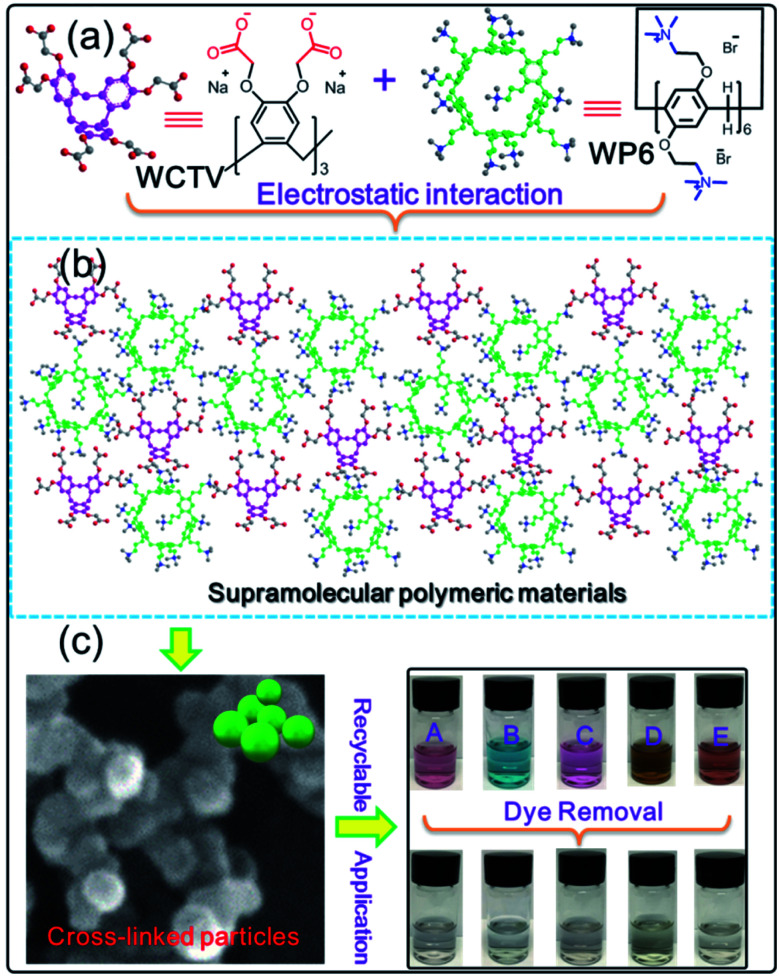
(a) Chemical structures and cartoon representations of WP6 and WCTV. (b) The formation of supramolecular polymer networks based on WP6 and WCTV (WP6&WCTV). (c) TEM image of WP6&WCTV and their application in removing dyes from water.

To obtain the WP6&WCTV polymeric material, WP6 (10^−4^ M) and WCTV (2 × 10^−4^ M) were dissolved in water respectively. Then the solution of WP6 and WCTV was dropped into water with a molar ratio of 1 : 2 under vigorous stirring simultaneously. WP6&WCTV was obtained as gray powders after washing with distilled water and CH_3_OH twice. It was found that WP6&WCTV was completely insoluble in common organic solvents (Fig. S13†), indicating the formation of cross-linked structures. Fig. S6† shows the FT-IR spectra of WP6, WCTV and WP6&WCTV. The spectrum of WP6&WCTV showed the overlap of WP6 and WCTV, confirming the crosslinking between WP6 and WCTV. Then the obtained WP6&WCTV was verified with solid-state ^13^C-NMR. The ^13^C-NMR spectrum of WP6&WCTV showed resonances at 172.8 ppm, corresponding to carbonyl carbons. The resonance peaks at 150.2 ppm, 147.1 ppm, 144.9 ppm, 132.8 ppm, and 114.3 ppm correspond to the sp^2^ carbons of the phenyl rings. The peaks at 66.1 ppm, 54.7 ppm, 36.5 ppm and 30.2 ppm correspond to methylene carbons near carbonyl groups, methyl carbons near N atoms, and bridged methylene carbons, respectively (Fig. 1a). In addition, as shown in Fig. 1b, WP6&WCTV exhibited excellent stability up to 270 °C as investigated by thermogravimetric analysis (TGA).

**Fig. 1:**
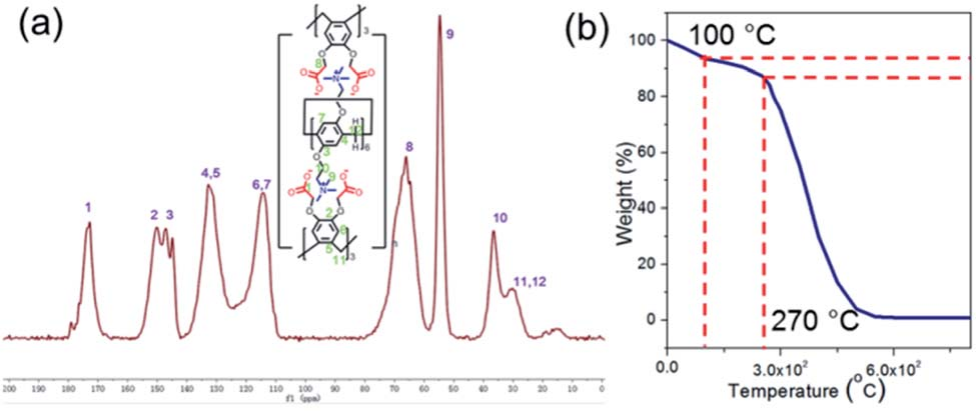
(a) Solid ^13^C NMR spectrum of WP6&WCTV. (b) Thermogravimetric analysis of WP6&WCTV.

The morphology of 3D polymeric WP6&WCTV materials was then investigated by scanning electron microscopy (SEM) and transmission electron microscopy (TEM). As shown in Fig. 2a, 3D cross-linked particles with a diameter of about 200 nm were observed, and this was also confirmed by the TEM image (Fig. 2b). Additionally, elemental analysis of WP6&WCTV showed that it consisted of 5.31% N, 63.49% C, and 7.18% H (Table S1†). Furthermore, energy dispersive X-ray analysis (EDX) mapping analysis (Fig. 2c) confirmed the homogeneous distributions of C, N, and O across an enlarged micro-particle; the analysis provided evidence that the polymeric materials were composed of WP6 and WCTV through electrostatic interactions with Br^−^ and Na^+^ removed. Unfortunately, we failed to determine the surface area of WP6&WCTV due to its *S*_BET_ being almost zero, which is in contrast to common porous materials. The extremely low surface area might due to the spherical morphology of WP6&WCTV. However, the cavity of pillar[5]arene incorporated into this 3D polymeric material provided an ideal platform to complex organic dye from water through host–guest interactions.

**Fig. 2:**
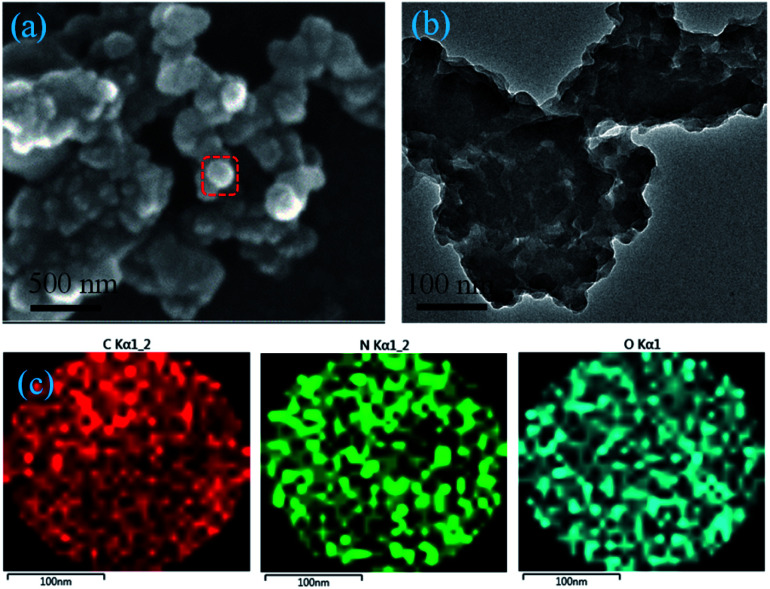
(a) SEM image of the WP6&WCTV materials. (b) TEM image of the WP6&WCTV materials. (c) EDX mapping of the WP6&WCTV materials (color code: C, red; N, green; O, cyan).

To test the performance of WP6&WCTV in organic pollutant removal, we evaluated five dyes causing harm to the environment: azophloxine (AX), ingrain blue (IB), rhodamine B (RhB), basic brown (BB) and alizarin red (AR). We submersed 5.00 mg WP6&WCTV materials into an aqueous solution of organic pollutants (0.100 mM, 5.00 mL) for 30 min and found that the dye solution became colorless (Fig. 3, inset). Then we used UV-Vis absorbance spectra to investigate the dye removal efficiency. As shown in Fig. 3, the characteristic peaks of each organic dye decreased sharply with absorbance time, indicating the dye concentration decreased substantially. It can be seen that the adsorption can reach equilibrium within 30 min, indicating a rapid adsorption process. All five dyes can be efficiently removed from water with uptakes greater than 85% (Table S2†). Importantly, WP6&WCTV materials can almost remove BB (97.3%) completely from water in 30 min (Fig. 3b). This is because the aniline groups on BB molecules well match the cavity of pillar[6]arene.^12^ These results confirmed that the WP6&WCTV polymeric material is an ideal platform for organic dye removal from water. Further ^1^H NMR investigation showed that the host–guest interactions between the host (WP6 or WCTV) and the organic dye caused this adsorption (Fig. S11–S15†).

**Fig. 3:**
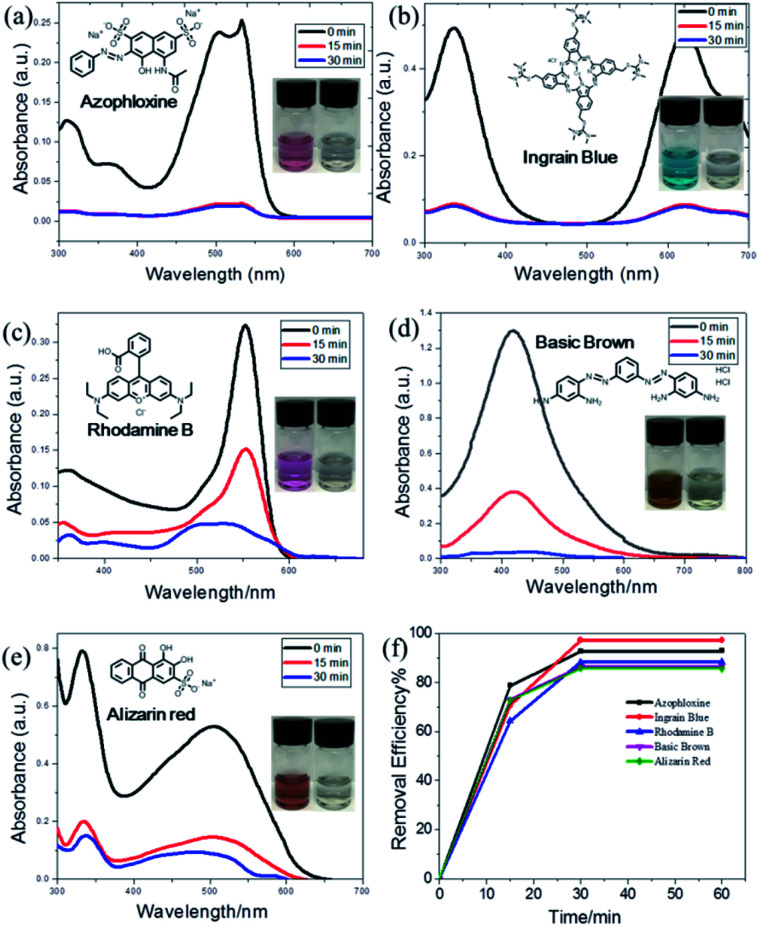
Time-dependent UV-Vis spectra of (a) azophloxine, (b) ingrain blue, (c) rhodamine B, (d) basic brown and (e) alizarin red solutions in the presence of WP6&WCTV materials. (f) Time dependence of dye removal efficiency. The insets show photos of the dye solutions before and after addition of WP6&WCTV materials.

Reusability is an important property of adsorbent materials. We also investigated the reuse ability of the obtained WP6&WCTV polymeric materials. The used WP6&WCTV was filtered and washed with water under ultrasonication and then dried under vacuum. The dried WP6&WCTV was added to the aqueous IB solution, which was then stirred for 30 min. As shown in Fig. 4, the IB removal efficiency was calculated to be 92% even after three cycles, indicating that WP6&WCTV had been regenerated successfully. With the low synthetic cost and good reusability, WP6&WCTV is economical and practical for waste-water treatment.

**Fig. 4:**
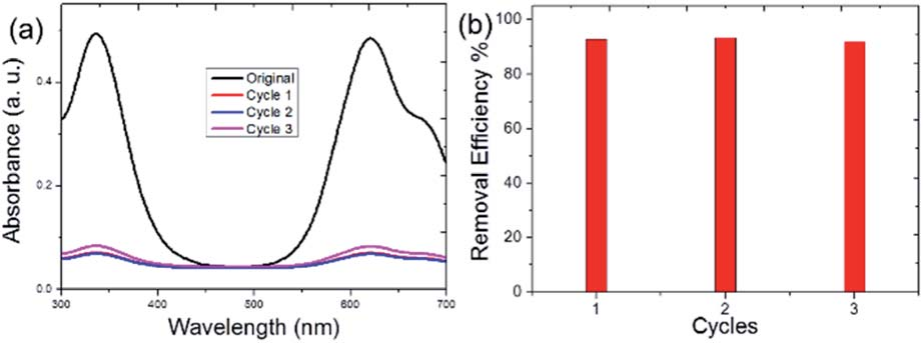
Evaluation of WP6&WCTV regeneration with IB as a model dye: (a) UV-Vis spectra of IB solutions and (b) removal efficiencies calculated on the basis of the decrease in absorption intensity.

## Conclusions

In conclusion, a new adsorbent material (WP6&WCTV) based on water soluble pillar[6]arene and cyclotriveratrylene was constructed successfully *via* electrostatic interactions. Its composition and 3D morphology were fully characterized by solid-state ^13^C-NMR, SEM, TEM and EDX mapping studies. Specifically, the obtained WP6&WCTV can be used as an adsorbent material to remove organic dye from water. In addition, this material can be regenerated easily by using a simple washing procedure with no loss in performance. These excellent findings demonstrate that this pillar[6]arene-based 3D network polymer can contribute to the removal of a wide range of micro-pollutants during water and waste-water treatment. We hope WP6&WCTV can be applied in real life fields, such as chemical separation and wastewater treatment.

## Conflicts of interest

There are no conflicts to declare.

## Supplementary Material

NA-003-D0NA00938E-s001
