# Supplementary material for: Pillar[6]arene-based supramolecular polymeric materials constructed via electrostatic interactions for rapid and efficient organic dye removal from water
Source: Nanoscale Adv. 2021 Feb 15;3(7):1906–9. doi: 10.1039/d0na00938e (PMC9417795; doi:10.1039/d0na00938e)
Supplement: NA-003-D0NA00938E-s001 [file NA-003-D0NA00938E-s001.pdf]

Electronic Supplementary Material (ESI) for Nanoscale Advances.
This journal is © The Royal Society of Chemistry 2021

Pillar[6]arene-based supramolecular polymeric material constructed via electrostatic interaction for rapid and efficient organic dye removal from water

Xin Yan,[a,†] Youyou Huang,[a,†] Moupan Cen,[a] Jin Wang,[a] Jian Shi,*[,b] Bing Lu,[a] Yang Wang,[a] Yong Yao*[,a]

[a]*School of Chemistry and Chemical Engineering, Nantong University, Nantong, Jiangsu, 226019, P.R. China.*

[b]*Nantong University Anlysis&Testing Center, Nantong University, Nantong, Jiangsu, 226019, P. R. China.*

E-mail: shi.j1@ntu.edu.cn; yaoyong1986@ntu.edu.cn

[†]*These authors contributed equally to this work.*

Supporting Information (12 pages)

1.	*Materials and methods*	S2
2.	*Synthesis of* ***WP6***	S3
3	*Synthesis of* ***WCTV***	S4
4.	*Characterization of* ***WP6&WCTV***	S7

1. Materials and methods

All reagents were commercially available and used as supplied without further purification. Deuterated solvents were purchased from Cambridge Isotope Laboratory (Andover, MA). **WP6** was prepared according to the published procedure.[S1] **WCTV** was prepared according to the published procedure.[S2] NMR spectra were recorded on a Varian

Unity 500 MHz spectrometer. Mass spectra were recorded on a Micromass Quattro II triple-quadrupole mass spectrometer using electrospray ionization with a MassLynx operating system. Transmission electron microscopy (TEM) investigations were carried out on a Hitachi Zoom-1 HC-1 instrument. SEM was carried out with a Hitachi S-4800 field emission scanning electron microscope. Solid-state nuclear magnetic resonance (NMR) spectra were recorded on a BRUKER 400WB AVANCE III spectrometer. Fourier transform infrared spectroscopy (FT-IR) spectra were recorded on a Thermo Nicolet iS10 spectrometer. Elemental analyses were carried out on a Vario EL Cube instrument. Thermogravimetric Analysis (TGA) was carried out on a DSCQ1000 Thermal Gravimetric Analyzer. UV–vis spectra were taken on a PerkinElmer Lambda 35 UV–vis spectrophotometer.

2. Synthesis of WP6

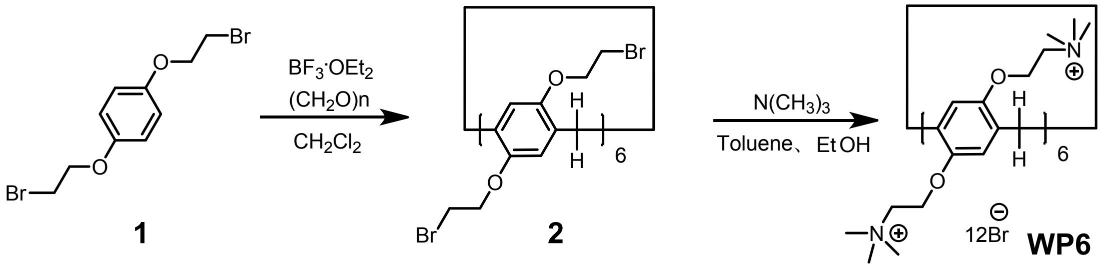

Scheme S1. Synthetic route of **WP6**.

Compound **2** was prepared according our previous report.[S1] **1** (4.86 g, 15 mmol), paraformaldehyde (0.69 g, 23 mmol), were stirred in 250 mL dry $CHCl_3$, then 3.26g BF_3•Et_2O was added. The reaction mixture was stirred at r.t. for 1 h, and then 250 ml water was added. The organic layer was collected and then the solvent was evaporated to get crude product. The crude product was purified by flash column chromatography on silica gel (CH_2Cl_2/PE, *v*/*v* 1:1) to give **2** as a white solid (0.62 g, yield: 12 %).

2 (1 g, 3 mmol), and 1.05 g of trimethylamine were stirred in 50 mL

CH_3CH_2OH/Toluene mixture at 100 °C for 8 h, then the solvent was evaporated to get crude product. The crude product was washed with $CHCl_3$ three times to give **WP6** as a white solid (1.58 g, yield: 95 %). The ^{1}H NMR spectrum of WP6 is shown in Figure 1a.^{1}H NMR (400 MHz, D_2O) δ 6.83 (s, 12H), 4.45-4.36 (m, 24H), 3.81-3.72 (m, 36H), 3.20-2.94 (m, 108H)。

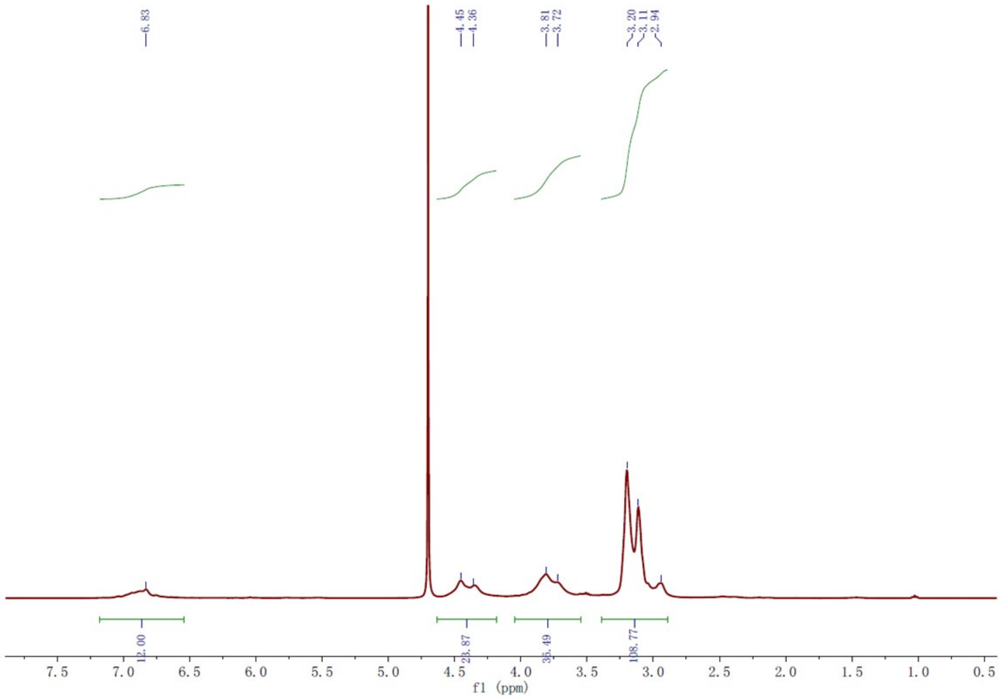

Figure S1a. ^{1}H NMR spectra of **WP6** in D_2O at 400 MHz.

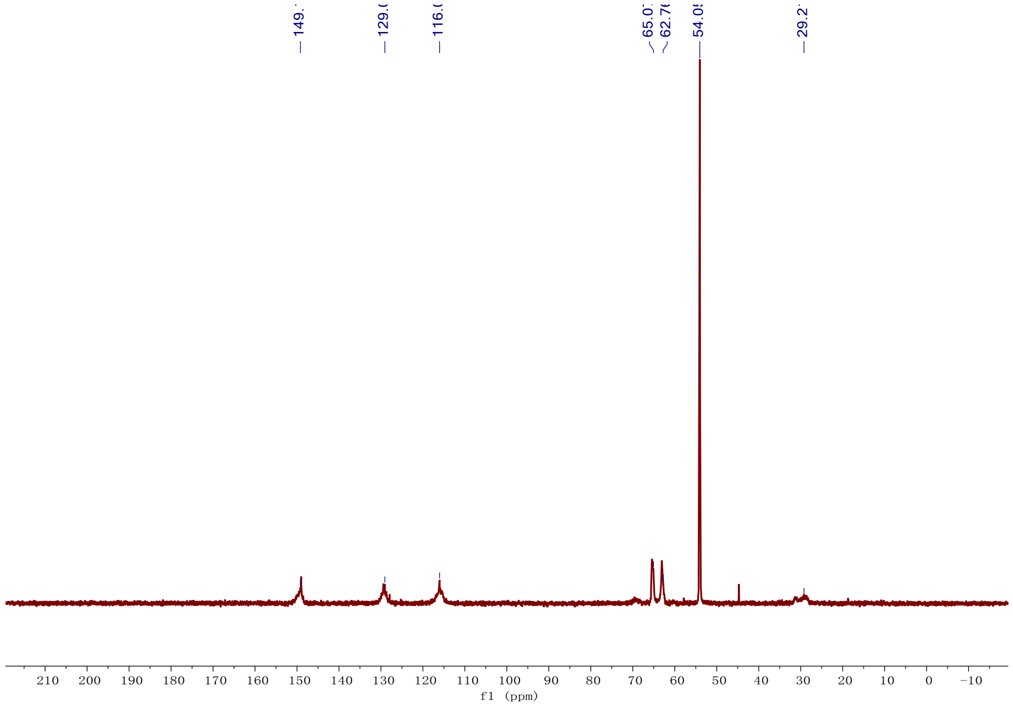

Figure S1b. ^{13}C NMR spectra of **WP6** in D_2O at 100 MHz.

3. Synthesis of WCTV

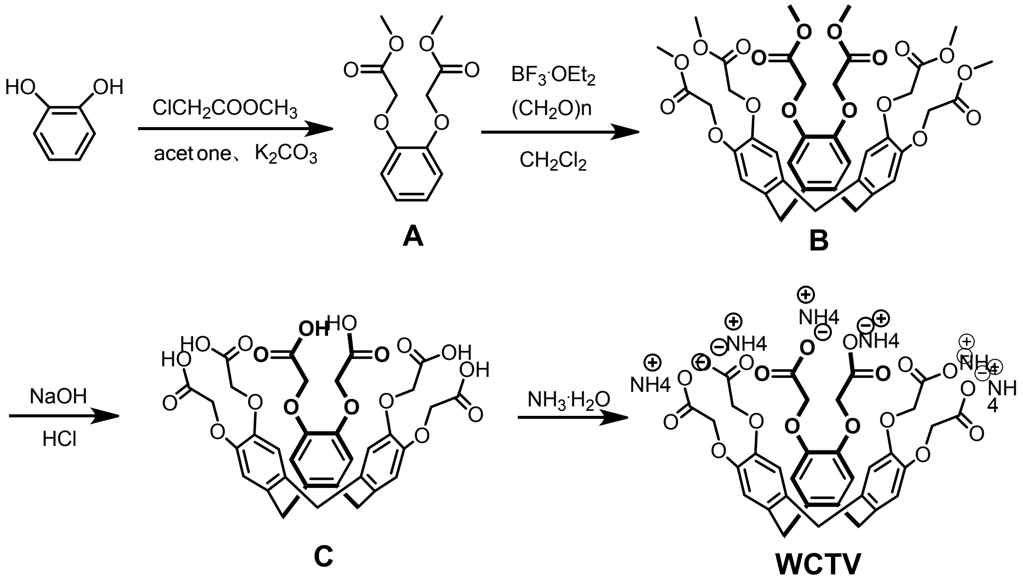

Scheme S2. Synthetic route of **WCTV**.

A mixture of Catechol (5 g, 45 mmol), methyl chloroacetate (14.86 g, 136 mmol), and K_2CO_3 (9.5 g, 68.8 mmol) was refluxed in the presence of N_2 in 200 mL acetone overnight. After the reaction was completed, the mixture was concentrated and recrystallized from methanol to obtain Compound **A** (10.6 g, yield: 92%) as a pale yellow solid.

The ^1H NMR spectrum of Compound **A** is shown in Figure 2.^1H NMR (400 MHz, Chloroform-*d*) δ 6.92 (ddd, *J* = 27.0, 6.1, 3.6 Hz, 4H), 4.73 (s, 4H), 3.79 (s, 6H)。

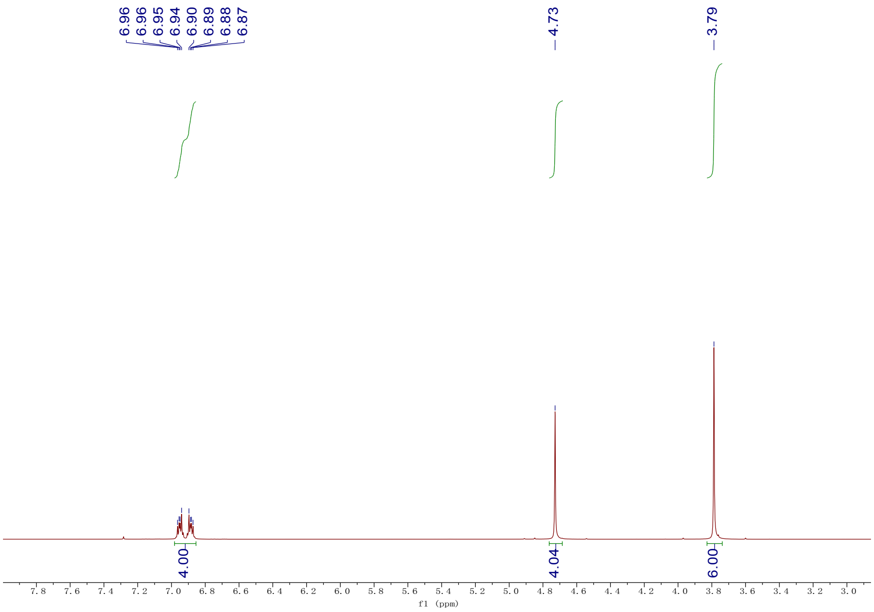

Figure S2. ^{1}H NMR spectra of **A** in $CDCl_3$ at 400 MHz.

A (2.92 g, 11.5 mmol) and Paraformaldehyde (0.698 g, 23.0 mmol) were added to 200 mL CH_2Cl_2, BF_3•Et_2O was added to the mixture, stirred at r.t. for 3 h, then methanol (200 mL) was added to give crude product as white solid. Compound B was collect by vacuum filtration, recrystallized in CH_2Cl_2 / methanol mixture and dried in vacuum to give B (1.69 g, yield: 44%) as acicular crystal.

The ^{1}H NMR spectrum of Compound **B** is shown in Figure 3.^{1}H NMR (400 MHz, Chloroform-*d*) δ 6.86 (s, 6H), 4.71 (s, 12H), 4.65 (d, J = 13.8 Hz, 3H), 3.77 (s, 18H), 3.49 (d, J = 13.9 Hz, 3H)。

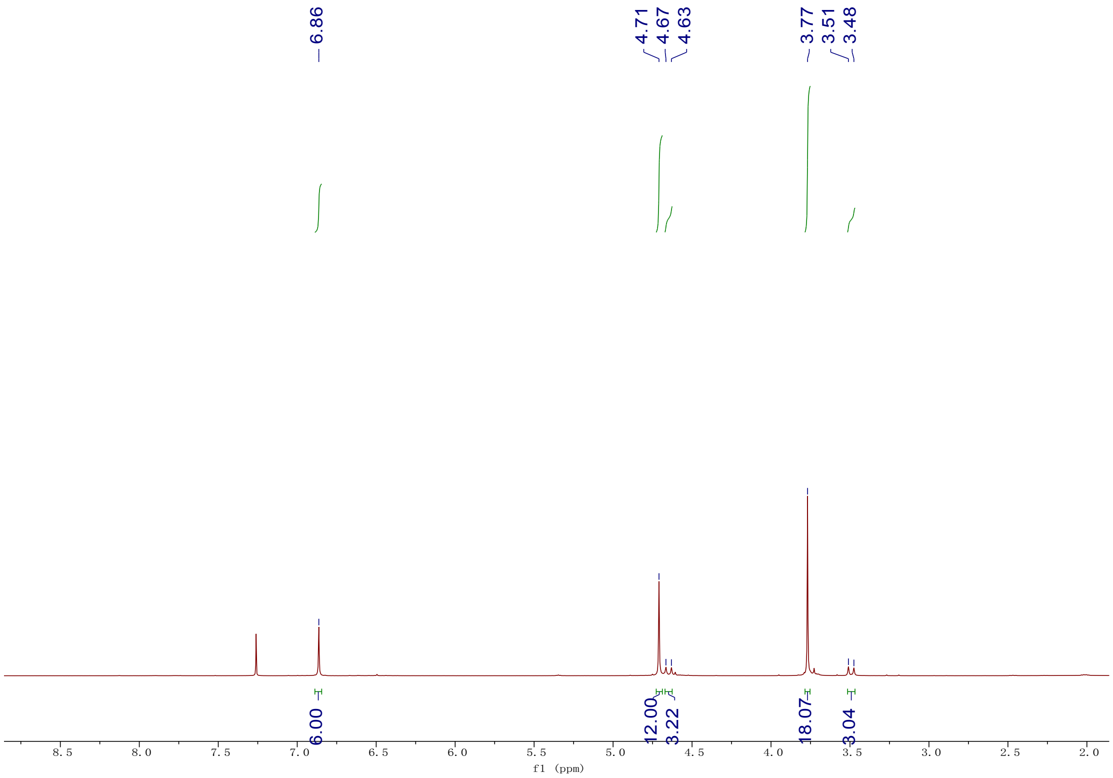

Figure S3. ^{1}H NMR spectra of **B** in $CDCl_3$ at 400 MHz.

B (1.20 g, 1.50 mmol) was stirred in 120 mL ethanol, added 120 mL 40% NaOH aqueous solution and refluxed for 10 h. The reaction solution was concentrated under reduced pressure, diluted with 30 mL of water, and then acidified with HCl. The precipitate was collected by filtration, then washed the precipitate with water to give C (0.998g, yield: 93%).

The ^{1}H NMR spectrum of Compound **C** is shown in Figure 4. ^{1}H NMR (400 MHz, DMSO-d_6) δ 6.97 (s, 6H), 4.66 (s, 6H), 4.61 (s, 12H), 3.42 (d, J = 13.4 Hz, 6H)。

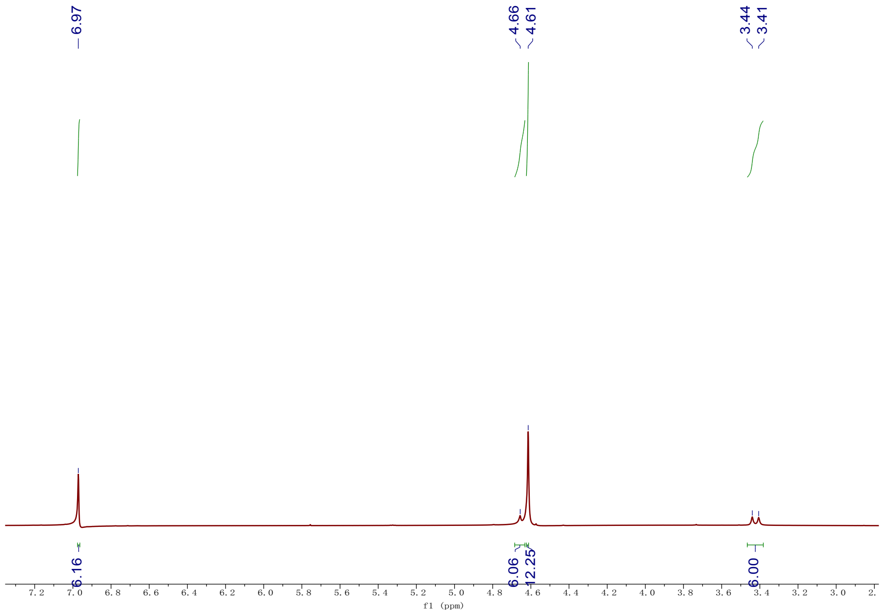

Figure S4. ^1H NMR spectra of **C** in DMSO at 400 MHz.

C (0.900 g, 1.25 mmol) was stirred in 40% $NH_3 \cdot H_2O$ aqueous solution under reflux for 5 h, then the reaction solution was concentrated under reduced pressure and filter to give the crude product. The crude product was washed with ethanol 3 times and then dried in vacuum to give WCTV (1.03 g, yield: 99%) as white solid.

The ^1H NMR spectrum of Compound **WCTV** is shown in Figure 5. ^1H NMR (400 MHz, Deuterium Oxide) δ 6.80 (s, 6H), 4.58 (s, 3H), 4.38 (s, 12H), 3.42 (d, J = 13.8 Hz, 3H)。

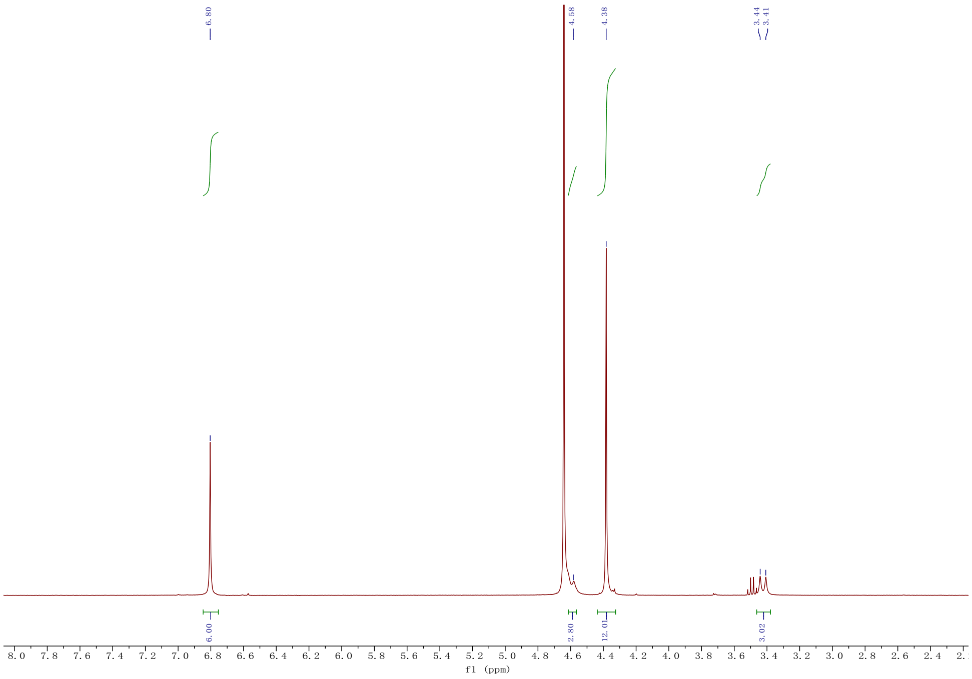

Figure S5. ^1H NMR spectra of **WCTV** in D_2O at 400 MHz.

4. Characterization of WP6&WCTV

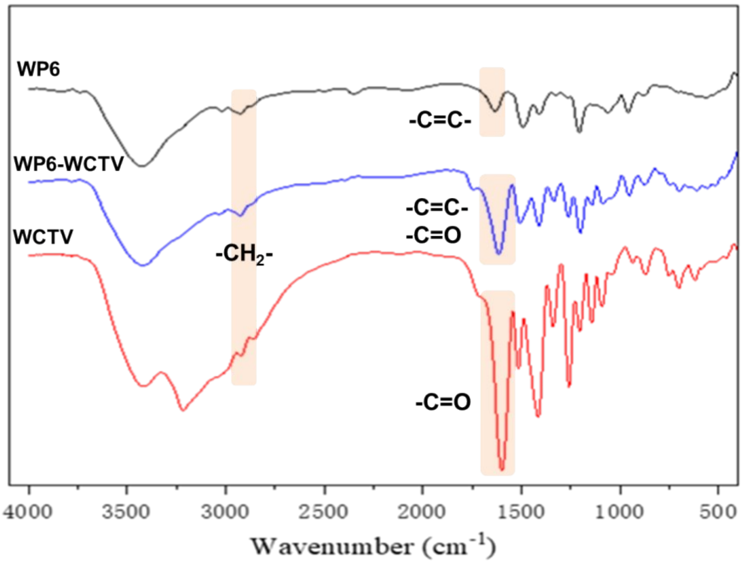

Figure S6. FT-IR spectra of **WP6**, **WP6&WCTV**, and **WCTV**.

Table S1 Elemental analysis data of polymeric **WP6&WCTV**.

	Name	Weight	N %	C %	H %
1	**WP6&WCTV**	1.9877 mg	5.26	63.41	7.16
2	**WP6&WCTV**	1.9932 mg	5.33	63.59	7.19
3	**WP6&WCTV**	1.9968 mg	5.34	63.46	7.20

Table S2. The Removal Efficiency of the dye

Dye	Removal Efficiency %
Azophloxine	86.5
Ingrain Blue 1	92.7
Rhodamine B	88.3
Basic Brown	97.3
alizarin red S	85.9

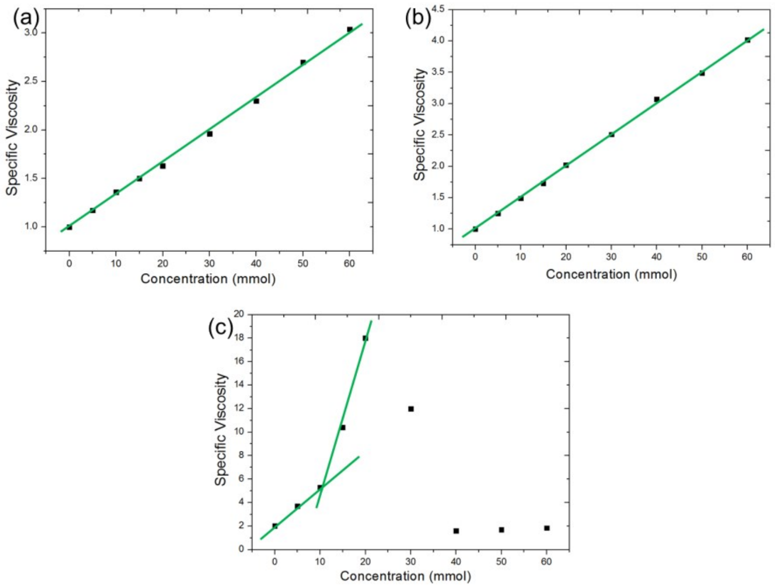

Fig S7 Specific viscosity of (a) **WCTV**, (b) **WP6** in water versus the concentration. (c) When fixed **WP6** = 20 mmol, specific viscosity of the system versus the concentration of **WCTV**.

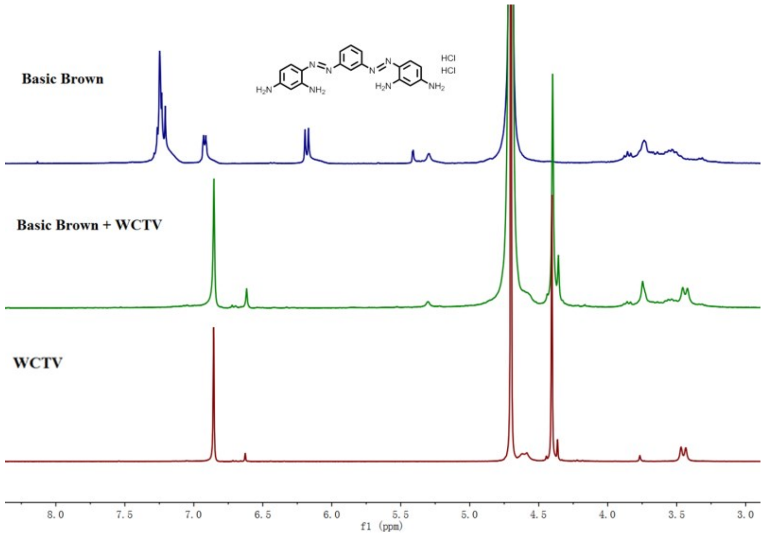

Fig. S8 ^1H NMR studies of Basic Brown and WCTV in D_2O.

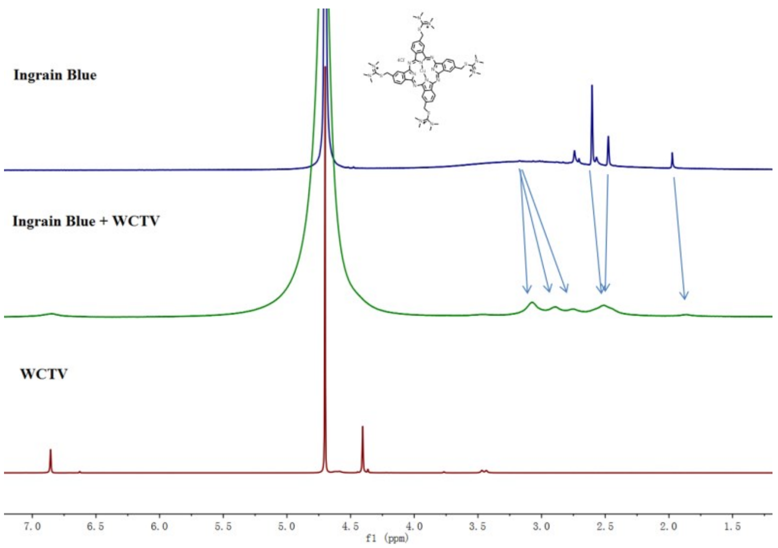

Fig. S9 ^1H NMR studies of Ingrain Blue and WCTV in D_2O.

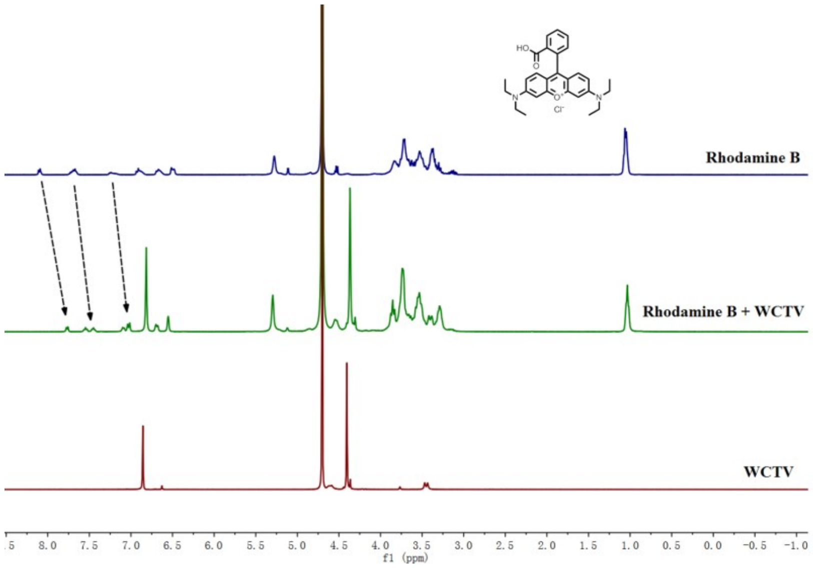

Fig. S10 ^1H NMR studies of Rhodamine B and WCTV in D_2O.

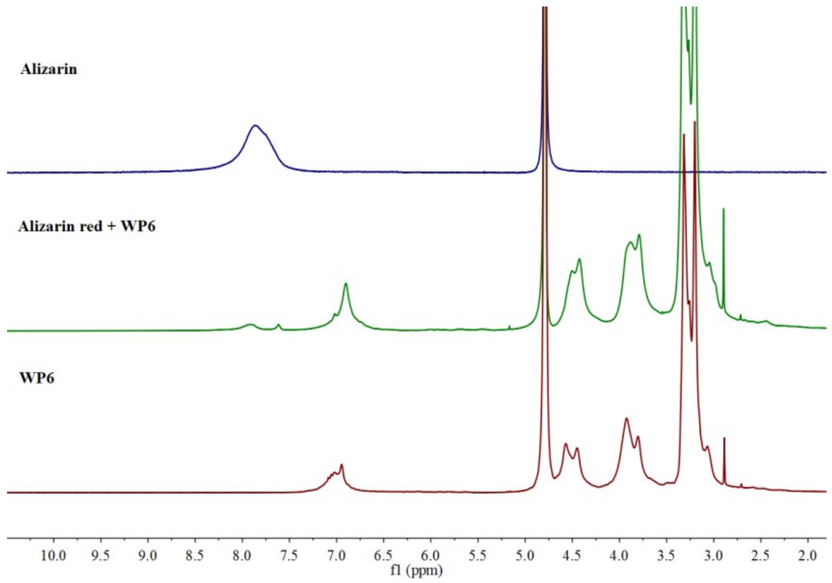

Fig. S11 ^{1}H NMR studies of Alizarin Red and WP6 in D_2O.

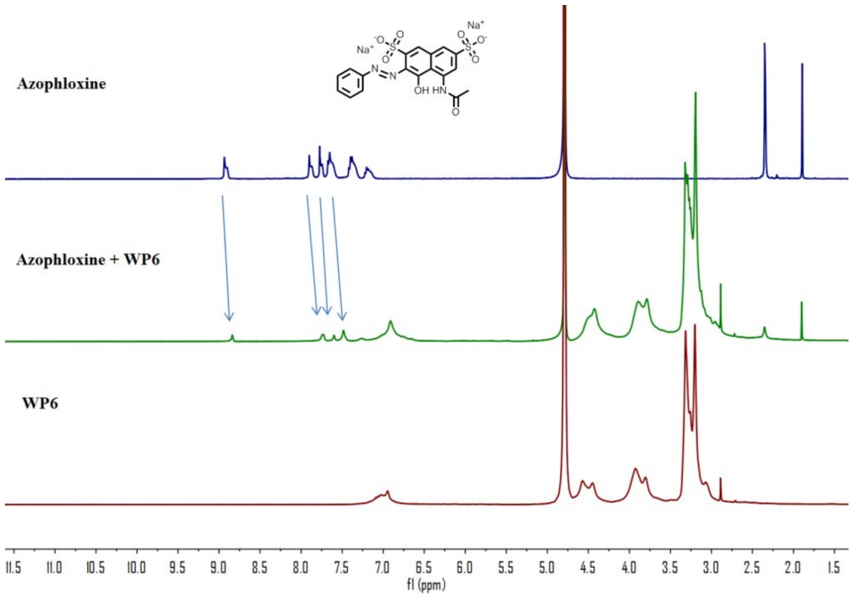

Fig. S12 ^{1}H NMR studies of Azophloxine and WP6 in D_2O.

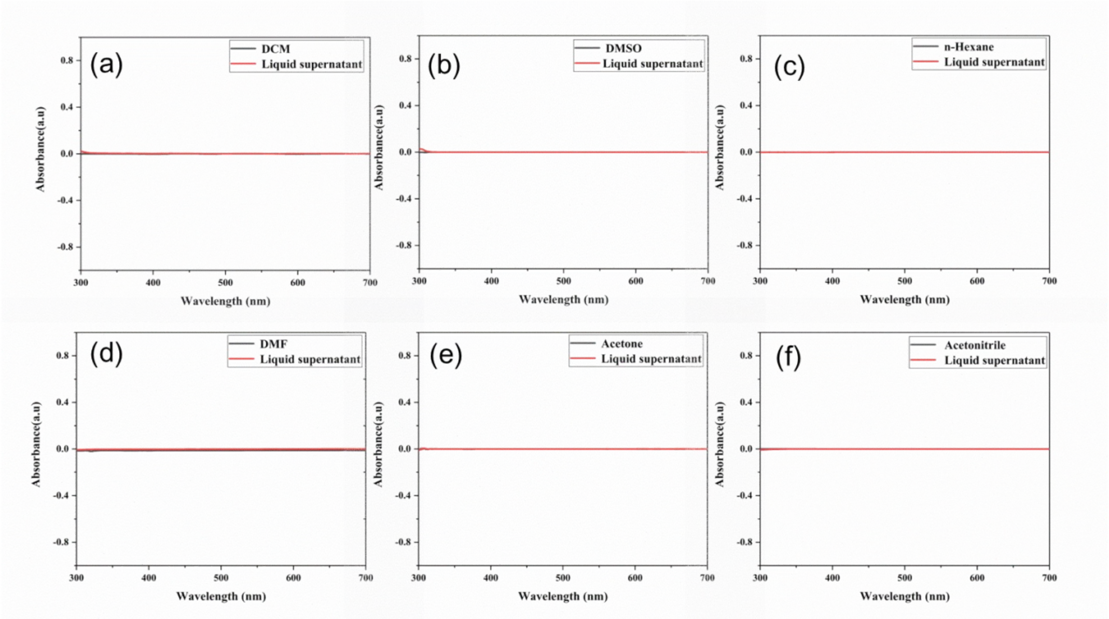

Fig. S13 UV spectra of different pure organic solvents and after **WP6&WCTV** were dispersed in the solvents overnight.

Reference

S1. Y. Yao, J. Li, J. Dai, X. Chi, and M. Xue, *RSC Adv.*, **2014**, *4*, 9039-9043.

S2. D. Xia, Y. Li, K. Jie, B. Shi, and Y. Yao, *Org. Lett.*, **2016**, *18*, 12, 2910-2913.
